# Verbalising importance of supply chain management in access to health services

**DOI:** 10.1186/s40545-021-00352-5

**Published:** 2021-11-16

**Authors:** Abdul Ghaffar, Arash Rashidian, Wasiq Khan, Muhammad Tariq

**Affiliations:** 1 Alliance for Health Policy & Systems Research, World Health Organization, 20 Avenue Appia, Geneva, Switzerland; 2 WHO Regional Office for the Eastern Mediterranean, Cairo, Egypt; 3 USAID Global Health Supply Chain Program, Islamabad, Pakistan

Supply chain management plays an important role in equitable access to essential medicines and services in low and middle income countries (LMICs). In addition, the COVID-19 pandemic has highlighted that supply chain preparedness is key to saving millions of lives globally. Supply of essential medicines and services is a wide-ranging issue and includes manufacturing, forecasting, procurement, distribution and delivery functions [1]. Each of these stages can be affected by a number of health system building blocks, including financing, governance, service delivery and human resource factors, which may play a role either in hampering or enhancing access. Here, we note that supply chains are embedded in health systems and suggest that strategies that prioritise equity, proactivity and partnership-building are key to stronger supply chains for stronger health systems. In addition, we outline the need for a better understanding of supply chain issues.

Supply chain management is a key function of health systems, Access to essential medicines and services is a multifaceted phenomenon and each factor of the supply chain can be affected by various factors. These include (a) personal factors, such as perceived needs of individuals, households and communities; (b) financial factors, such as pricing and out-of-pocket costs of medicines; (c) organisational factors, such as those differentially affecting primary, secondary and tertiary levels of care; (d) service delivery factors, such as availability and distribution; and (e) appropriate medicine utilisation factors, such as appropriate dispensing practices [2–4]. Health system planners and managers therefore must note that good supply chain management goes further than field health workers managing stock outs, or a resource distribution and delivery plan which is not divorced from overall system functions. Rather, it is an intrinsic part of the health system, which needs to be appreciated and recognised as an essential competency of relevant public health professionals.

The COVID-19 pandemic has exposed the limitations of both LMIC and high-income country health systems to respond to shocks [5, 6]. In early 2020, we noted countries struggling with their ability to reliably forecast, quantify and source PPE, ventilators and essential medicines, with consequent morbidity and mortality. It is likely that weaknesses in supply chain have contributed to morbidity and mortality during the pandemic. Supply chain management in response to the pandemic has been reactive, with a focus on short-term needs, rather than being proactive. Building stronger and more resilient health systems for the future will demand further investment in essential public health functions. In particular, enhancing disease surveillance systems which are fully embedded in health information systems may be helpful for forecasting and informing local manufacturing. Enhancing capacities of public health laboratories including their national distribution as well as establishment of strong national reference laboratories where they do not exist also must consider supply chain variables. Strengthening health systems post-pandemic presents an opportunity to systematically and sustainably address different supply chain bottlenecks that affect individual’s access to essential medicines and health care [7].

Supply chain management and health systems, before the COVID-19 pandemic, focussed on efficiency, provision of health care, and contribution to economic growth. We believe that post-COVID health systems and supply chains, recognising the needs and demands of individuals and societies, should put a higher emphasis on ensuring that equity and social determinants of health are addressed in the ongoing response to the pandemic and subsequent recovery, as these play a major role in ensuring the optimisation of investments and access to health services. We understand that significant investments have been made in health systems to improve access to essential medicines and services and strengthen supply chain systems. Indeed, this value of equity-oriented policies should be capitalised upon when designing and implementing supply chain management strategies post-pandemic. We believe that good public policy in relation to supply chain management cannot relate to people and communities adequately in LMICs without partnering with relevant industries. In interdependent economies of the world, there is a need to not only push intra-country supply chain preparedness, but also north-to-south and south-to-south linkages may be beneficial for the gains of industrial equity and socially just systems.

A significant body of literature exists on social, economic and geographic determinants of access to essential medicines and health services. However, the intersections between the sciences of health services, supply chain and surveillance systems are less well understood. Unfortunately, supply chain management is also historically an under-researched area of work in medicine and public health [8] which means best practices are not well documented and evaluated, and local innovations are harder to assess and share among countries. An underpinning cause is that “supply chain management” is not a subject that has been adequately taught in schools of public health, medicine and pharmacy. Sadly this has not been a priority for most planners and policymakers and has been left to the ability and capability of field workers, who have training in medicine and public health, and use their common sense or experiential learnings to manage the supply systems. However, supply chain difficulties during COVID-19 and associated morbidity and mortality have highlighted the faults in this approach and made evident the significance of supply chain management in contributing to access to essential medicines and health services. This situation demands the immediate attention of policymakers, funders, and academics. We propose that countries focus on building equity-oriented, sustainable supply chain mechanisms and invest in strengthening the capacities and knowledge for supply chain management through research and formal training of public health practitioners on supply chain issues [9]. This will surely facilitate progress towards Universal Health Coverage [10].
